# Varying Expression of Mu and Kappa Opioid Receptors in Cockatiels (*Nymphicus hollandicus*) and Domestic Pigeons (*Columba livia domestica)*

**DOI:** 10.3389/fgene.2020.549558

**Published:** 2020-10-15

**Authors:** Samantha L. Fousse, Bryce M. Golsen, David Sanchez-Migallon Guzman, Joanne R. Paul-Murphy, Joshua A. Stern

**Affiliations:** ^1^Department of Medicine and Epidemiology, School of Veterinary Medicine, University of California – Davis, Davis, CA, United States; ^2^College of Veterinary Medicine, University of Georgia, Athens, GA, United States

**Keywords:** opioid receptors, avian, gene expression, *OPRM1*, *OPRK1*, analgesia, qPCR

## Abstract

Avian species have varying analgesic responses to opioid drugs. Some of this variability could be due to extrinsic factors such as administration route or dose. However, intrinsic factors such as gene expression or polymorphic differences in opioid receptors may be important components.

**Objectives:**

The objectives of this study were to determine the relative gene expression and polymorphisms present for mu and kappa opioid receptors (*OPRM1* and *OPRK1*) in the cerebrum, brainstem, spinal cord, and footpad of cockatiels and pigeons.

**Methods:**

Tissue biopsies were obtained from 11 adult cockatiels (6 male and 5 female) and 11 adult pigeons (6 male and 5 female). RNA was extracted and qPCR was performed to determine the level of gene expression for *OPRM1* and *OPRK1* relative to a reference gene phosphoglycerate kinase 1 (*PGK1)* using the ΔΔCt method. Sanger sequencing was performed to identify polymorphisms, if present.

**Results:**

There were higher expression levels of *OPRM1* compared to *OPRK1* in all tissues examined regardless of species (*p* < 0.001, FDR *p* < 0.001) Cockatiels had less *OPRK1* expression in the cerebrum compared to pigeons (*p* = 0.005, FDR *p* = 0.004). Cockatiels had more *OPRM1* expression in the brainstem (*p* = 0.045, FDR *p* = 0.029), but less *OPRM1* expression in the footpad compared to pigeons (*p* = 0.029, FDR *p* = 0.021). No other significant differences in *OPRM1* or *OPRK1* expression were identified across species. Two missense polymorphisms were identified in *OPRK1;* none were found in *OPRM1.*

**Conclusion:**

The differential expression of opioid receptors between cockatiels and pigeons could have implications for variability in analgesic response between these two species.

## Introduction

Opioids are a diverse group of drugs that modify the transmission and perception of pain in vertebrates. Although three main classes of opioid receptors exist (mu, kappa, and delta), only drugs that act on mu and kappa receptors are frequently used in avian species (Hawkins and Paul-Murphy, 2011). The analgesic effects of opioids have a wide range of clinical efficacy depending on the avian species studied. For example, in cockatiels, mu agonists such as buprenorphine or hydromorphone did not yield a significant thermal antinociceptive effect when delivered intramuscularly (Guzman et al., 2018; Houck et al., 2018). However, similar doses of hydromorphone that were not effective in cockatiels induced thermal antinociception in another avian species, American kestrels (Guzman et al., 2013). The current opioids recommended for cockatiels are kappa agonists based on studies in other psittacine species and anecdotal clinical evidence (Paul-Murphy et al., 1999, 2009a, b; Sladky et al., 2006; Guzman et al., 2011, 2017).

It is possible that this lack of thermal antinociceptive response to hydromorphone and buprenorphine in cockatiels could be due to external factors such as dose, route of administration, and/or drug metabolism. For instance, orange-winged Amazon parrots did not have a thermal antinociceptive response to hydromorphone at 0.1 mg/kg, but did at 1 mg/kg (Guzman et al., 2017). This higher dose has not yet been evaluated in cockatiels.

Another explanation could be genetic differences in opioid receptor structure, distribution, or expression. In humans and other mammals, variable response to opioids can be due to polymorphisms that alter the expression or amino acid sequence of the opioid receptors (Lötsch and Geisslinger, 2005; Kadiev et al., 2008). The same may be true for avian species since genetically different strains of chickens have diverse responses to the same opioid administration (Hughes, 1990). The structure of mRNA, splicing differences, and sequence homology for opioid receptors was previously reported for three diverse avian species (Duhamelle et al., 2018). However, the relative tissue expression of mu and kappa receptors in healthy cockatiels has not yet been reported. Fortunately, a set of reliable qPCR reference genes across avian species has previously been developed (Olias et al., 2014). These reference genes along with the availability of avian-specific opioid receptor primer sets allow for a robust investigation into opioid receptor expression across species and tissue types (Khurshid et al., 2009; Duhamelle et al., 2018).

A side-by-side investigation into the expression of mu opioid (*OPRM1*) and kappa opioid (*OPRK1*) receptors in various tissues of two diverse avian species, *Nymphicus hollandicus* and *Columba livia domestica*, is beneficial to determine if opioid expression or polymorphic differences exist that may explain the variability of analgesic response seen in cockatiels. The pigeon represents an ideal species for comparison since it is widely used in opioid research and can discriminate between kappa opioid agonists and mu opioid agonists (Picker and Dykstra, 1989). This study is limited to mu and kappa opioid receptors since those are the opioids currently used and actively researched in the field of avian analgesia (Hawkins and Paul-Murphy, 2011).

### Objectives

The objectives of this study were to identify polymorphisms and compare the relative expression levels for *OPRM1* and *OPRK1* mRNA using real-time PCR analysis for tissue biopsies harvested from cerebrum, brainstem, spinal cord and footpad in two avian species. These tissues were selected to represent different regions of the opioid receptor pathway and have been shown to be present in avian tissues previously (Duhamelle et al., 2018). Based upon previous clinical and research observations, we hypothesized that cockatiels will have higher *OPRK1* expression compared *OPRM1* expression in various tissues. Additionally, we hypothesized that cockatiels will have higher *OPRK1* expression and lower *OPRM1* expression compared to pigeons.

## Methods

### Animals and Ethical Information

This study used 11 (6 male and 5 female) adult domestic pigeons (*Columba livia domestica)* and 11 (6 male and 5 female) adult cockatiels (*Nymphicus hollandicus*). Cockatiels were from the research colony of the Animal Science Department at the University of California – Davis. Pigeons were purchased from an authorized campus vendor.^1^ All birds were determined to be healthy based on physical examination prior to euthanasia and tissue collection. Tissue collection occurred on May 12, 2017. Birds were euthanized with intravenous administration of 100 mg/kg IV pentobarbital which adheres to the 2013 American Veterinary Medical Association Guidelines for avian euthanasia (Leary et al., 2013). Protocols have been approved by the Institutional Animal Care and Use Committee at the University of California – Davis (IACUC #20331).

### Sample Collection and Processing

Biological samples were rapidly dissected immediately after death using a separate sterile 4mm punch biopsy instrument for each sample. Punch biopsies were taken from the caudal cerebrum (telencephalon), brainstem (medulla oblongata), spinal cord (cervical and thoracic region), and center of the footpad with skin. The punch biopsy sample was immediately placed in 3mL of RNA*later*^TM^ RNA stabilization reagent (Invitrogen Ambion) and stored at 4C for 48–72 h, after which the RNA*later*^TM^ solution is decanted and tissue samples are stored at −80°C. Samples remained at −80°C until all samples of a single tissue type could be extracted at the same time. No tissues types were stored for <1 month or >1 year prior to extraction.

### RNA Extraction

All steps of the experiment from tissue storage to analysis were performed in the investigator’s laboratory (JS). The cerebrum, brainstem, and spinal cord samples were homogenized with the Bullet Blender Tissue Homogenizer (Next Advance, Troy, NY, United States). Tissue biopsy was placed in a tube containing Buffer RLT from the RNeasy^®^ MiniKit (Qiagen, Hilden, Germany) and zirconium oxide beads weighing twice the mass of the tissue sample. The tissue was processed at full speed for 5 min, repeatedly, until completely homogenized.

Due to the fibrous nature of the footpad tissue, the footpad tissue sample was homogenized with the 1600 MiniG^®^ (SPEX Sample Prep, Metuchen, NJ, United States). The footpad tissue biopsy was placed in a 5mL polyethylene tube SPEX Sample Prep, Metuchen, NJ, United States) containing a 3/8′′ 440-stainless steel grinding ball (OPS Diagnostics, Lebanon, NJ, United States). This sample was processed at 1,000 rpm for 2 min, refrozen in liquid nitrogen, and processed again at 1,000 rpm for 2 min. The tissue was combined with Buffer RLT from the RNeasy MiniKit and processed at 1,000 rpm for 1 min repeatedly until homogenized.

Lysate from both homogenization procedures was used for RNA extraction using the RNeasy^®^ MiniKit with the exception of spinal cord which was extracted with the RNeasy^®^ Fibrous Tissue Kit (Qiagen, Hilden, Germany). RNA quantity and purity were assessed on the NanoDrop Spectrophotometer (ThermoFisher Scientific). The Quantitech^®^ Reverse Transcription Kit (Qiagen, Hilden, Germany) was used to remove genomic DNA contamination if present and convert 100 ng of RNA to cDNA using the kits random hexamer primers. The cDNA was stored at −20°C until use in qPCR.

### qPCR Primers

The primer sets used were previously published for avian species (Table 1; Khurshid et al., 2009; Olias et al., 2014; Duhamelle et al., 2018). The reference genes paired with the original opioid receptor primer set were tested if the primer sequences were available (GAPDH and ACTB) (Duhamelle et al., 2018). Additionally, a previously published reference gene, PGK1, that was stably expressed, specific, and similar in size to the opioid products was tested (Olias et al., 2014). All qPCR primers were validated using a melting curve analysis on pooled cDNA from both pigeons and cockatiels for all tissue types to ensure that a single product was amplified (Supplementary Figure 1). If there was not a single melting curve, those primer sets were excluded from further analysis. The products were sequenced by the UC DNA Sequencing Facility using an ABI 3730 Capillary Electrophoresis Genetic Analyzer with ABI Big Dye Terminator v3.1 Cycle Sequencing chemistry. Sequences were aligned and polymorphisms identified with DNASTAR Navigator SeqManPro software. UCSC genome browser and NCBI Ensembl was used to determine position of polymorphisms in the *Columba livia* rock pigeon assembly (GCF_000337935.1) (Kent et al., 2002).

**TABLE 1:** Primer sets tested for qPCR.

Gene	Species	Location	Size (base pairs)	Primer set F 5′–3′ R 5′–3′	Efficiency
*OPRM1* (16)	GenBank: DV580289.2 **Taeniopygia guttata** (Zebra finch)	Exon 2	165	GCAGATGCCCTAGCAACAAG	1.04
				CACGTAGCGATCCACACTCA	
*OPRK1* (14)	*Amazona aestiva* (Blue-fronted Amazon Parrot) NCBI:txid12930	Spans intron 3	176	CACCTCTCAAGGCAAAGATAA	1.00
				ACAGATTTTCATGAAGATGTCCC	
*PGK1* (15)	*Gallus gallus* Chicken NM_204985	Spans intron 1	167	AAAGTTCAGGATAAGATCCAGCTG	0.98
				GCCATCAGGTCCTTGACAAT	
GAPDH (14)	*Amazona aestiva* (Blue-fronted Amazon Parrot) NCBI:txid12930	Unknown	177	GCCATTCCTCCACCTTTGATG	Excluded
				GCTGTGTGTTCGGCTCACTC	
ACTB (14)	*Amazona aestiva* (Blue-fronted Amazon Parrot) NCBI:txid12930	Unknown	200	CAACTGGGATGACATGGAGA	Excluded
				GCACAGCCTGGATGGCCAC	

### qPCR Protocol

The quantitative real-time polymerase chain reaction (qPCR) experiment was performed according to Minimum Information for Publication of Quantitative Real-time PCR Experiments (MIQE) guidelines (Bustin et al., 2009). The Rotor-Gene SYBR green PCR kit (Qiagen, Hilden, Germany) was used in conjunction with the Rotogene Q cycler (Qiagen, Hilden, Germany) to perform qPCR analysis. The protocols for the qPCR primers were optimized by increasing or decreasing forward and reverse primer concentration to ensure that efficiency of each primer set was between 0.9 and 1.1. For *OPRM1* amplification a 10 μL reaction volume was used (5 μL of 2X Buffer Master Mix, 0.5 μL of 5 ng/μL of forward and reverse primer, 3 μL of water, and 1 μL of cDNA). For OPRK1 amplification a 10 μL reaction volume was used (5 μL of 2X Buffer Master Mix, 0.5 μL of 10 ng/μL of forward and reverse primer, 3 μL of water, and 1 μL of cDNA). For *PGK1* amplification a 10 μL reaction volume was used (5 μL of 2X Buffer Master Mix, 0.5 μL of 10 ng/μL of forward and reverse primer, 3 μL of water, and 1 μL of cDNA). Cycling included a hold temp of 95C for 5 min, followed by 40 cycles of 95C for 5 s and 60°C for 10 s. Samples were run in triplicate. A no-template-control and a no reverse-transcription control was run on each plate. The analysis was performed using Rotor-Gene Q Series Software Version 2.1.0.

### Statistical Analysis

A sample technical triplicate was excluded if the standard deviation for the cycle threshold (Ct) values was greater than one. The following calculation of relative changes in gene expression was used: Δ*Ct* = *Ct*_*reference gene*_−*Ct*_*opioid receptor gene*_. The ΔΔCt was calculated as ΔΔ*Ct* = *average*Δ*Ct*_*pigeons*_−*average*Δ*Ct*_*cockatiels*_. Fold change was reported as 2^ΔΔ*Ct*^. For species comparisons, a fold change greater than one identifies relatively greater gene expression in pigeons, while a fold change less than one identifies relatively greater gene expression in cockatiels. For opioid receptor comparisons, a fold change greater than one identifies relatively greater gene expression in OPRM1 compared to OPRK1. The delta-delta CT values and fold change differences were determined using a standard formula worksheet (Mac Microsoft Office 365 Excel Version 16.20).

Statistical analysis was performed using *Prism8* (GraphPad Software, San Diego, CA, United States). A biological sample was excluded if identified as an outlier by the ROUT method (Q = 1%). To determine the influence of sex on gene distribution, a Shapiro-Wilk normality test was used to test for normality for sex in each tissue type. If both sexes had a normal distribution, an unpaired *t*-test was performed. If either sex did not pass normality, a Mann-Whitney test was performed.

To determine the effect of species on gene expression, the D’Augustino-Pearson Omnibus Normality Test was used to determine if the results were parametric. If both the cockatiel and pigeon sample set was parametric for a specific tissue type the ΔCt differences were tested using an unpaired *t*-test with results reported as (mean ± SD). If either the cockatiel and/or pigeon sample set was non-parametric for a specific tissue type the ΔCt differences were tested using a Mann-Whitney Test with results reported as (median ± inter quartile range). A statistically significant difference in ΔCt was set at a *p*-value less than 0.05. To correct for multiple testing, the False Discovery Rate (FDR) correction was utilized.

## Results

There were three reference genes tested: *PGK1, GAPDH*, and *ACTB*. *GAPDH* and *ACTB* were non-specific, while *PGK1* was specific (Supplementary Figure 1). The two opioid genes were also specific to a single product (Supplementary Figure 1). Sequencing of the single products confirmed that the expected gene was amplified (Supplementary File 1). There were 12 polymorphisms identified within the two opioid genes (Supplementary Table 1). None of the polymorphisms were in the primer binding region. Ten were predicted to be synonymous. Two were predicted to be a missense mutation in *OPRK1* resulting in an amino acid change from methionine in pigeons to either valine or isoleucine in cockatiels (Supplementary Figure 2).

**FIGURE 1:**
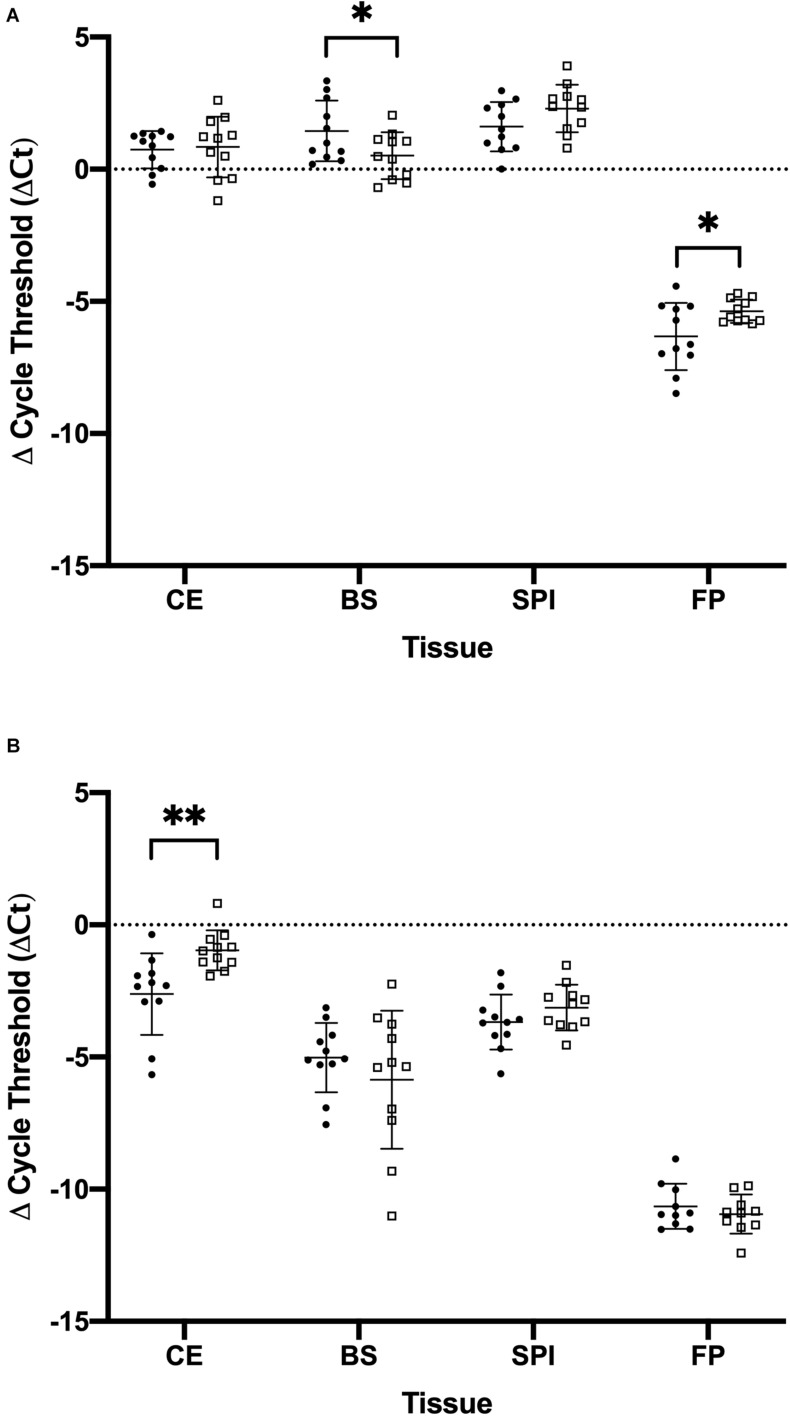
Comparison of the amount of mu opioid receptor (*OPRM1*) and kappa opioid receptor (*OPRK1*) gene expression in tissues of cockatiels and pigeons normalized to the reference gene phosphoglycerate kinase 1 (PGK1). **(A)** *OPRM1* **(B)** *OPRK1.* Mean and standard deviation are displayed. Parrots are closed circles. Pigeons are open squares. ^∗^*p* < 0.05; ^∗∗^*p* < 0.01.

The optimized cycle thresholds for *OPRK1*, *OPRM1*, and *PGK1* were 0.0996, 0.0136, and 0.3832, respectively. Kappa and mu opioid receptor mRNA were expressed in all tissues studied (Figure 1). There were no significant sex differences for the opioid receptors within each tissue type (Supplementary Table 2); therefore, the remaining data was analyzed with sexes combined (Supplementary Table 3). Data were normally distributed, and no outliers were detected, consequently all combined data was analyzed with a *t*-test. There was one pigeon and one cockatiel with *OPRK1* expression levels in the footpad below the limit of detection for this assay. There were significantly higher expression levels of *OPRM1* compared to *OPRK1* in all tissues examined regardless of species (Table 2). These differences remained significant after FDRcorrections.

**TABLE 2:** Fold change expression differences between *OPRM1* and *OPRK1* in cerebrum, brainstem, spinal cord, and foot pad of cockatiels and pigeons.

Species	Tissue	Fold-change	*P*-value	*P*-value (FDR)
Cockatiel	Cerebrum	10.25	<0.0001*	<0.0001
Pigeon	Cerebrum	3.49	0.0003*	0.0002
Cockatiel	Brainstem	88.54	<0.0001*	<0.0001
Pigeon	Brainstem	82.78	<0.0001*	<0.0001
Cockatiel	Spinal cord	39.02	<0.0001*	<0.0001
Pigeon	Spinal cord	42.99	<0.0001*	<0.0001
Cockatiel	Foot pad	20.03	<0.0001*	<0.0001
Pigeon	Foot pad	47.61	<0.0001*	<0.0001

*A fold change of greater than one identifies relatively greater gene expression in OPRM1 compared to OPRK1. An asterisk indicates statistically significant p-values.*

There was differential expression between cockatiels and pigeons for the opioid receptors tested (Table 3). The cockatiel cerebrum had 271% less *OPRK1* gene expression compared to pigeons (*p* = 0.005) (Figure 1B). The cockatiel brainstem had 49% more *OPRM1* gene expression compared to pigeon (*p* = 0.045) (Figure 1A). The cockatiel footpad had 141% less *OPRM1* gene expression compared to the pigeon (*p* = 0.029) (Figure 1A). All of these differences withstood FDR corrections (Table 3). No other statistically significant differences in *OPRK1 or OPRM1* expression for the cerebrum, brainstem, spinal cord, or footpad tissues were identified between cockatiels and pigeons (Table 3).

**TABLE 3:** Fold change expression differences between cockatiels and pigeons for *OPRK1* and *OPRM1* in the cerebrum, brainstem, spinal cord, and foot pad.

Primer set	Tissue	Fold-change	*P*-value	*P*-value (FDR)
*OPRK1*	Cerebrum	2.71	0.005*	0.0039*
*OPRK1*	Brainstem	0.50	0.352	0.1778
*OPRK1*	Spinal Cord	1.68	0.194	0.1055
*OPRK1*	Foot Pad	0.72	0.421	0.1984
*OPRM1*	Cerebrum	1.05	0.804	0.3553
*OPRM1*	Brainstem	0.49	0.045*	0.0289*
*OPRM1*	Spinal Cord	1.77	0.094	0.0554
*OPRM1*	Foot Pad	1.41	0.029*	0.0205*

*A fold change greater than one identifies relatively greater gene expression in pigeons, while a fold change less than one identifies relatively greater gene expression in cockatiels. An asterisk indicates statistically significant p-values.*

## Discussion

This study focused on one specific aspect that could influence drug response, expression of the drug receptor. Variation of opioid receptor expression exists between tissue types within an avian species and across avian species. This is not surprising since variation in opioid receptor expression has been found in other species such as humans and rodents (Uhl et al., 2002). Similar to humans, both the cockatiel and pigeon tissues in this study had relatively lower *OPRK1* expression levels compared to *OPRM1* in all tissues with expression data (Peng et al., 2012). However, the findings documenting the level of expression for each type of receptor were somewhat unexpected when considering clinical experience and the previous antinociception studies in cockatiels (Guzman et al., 2018; Houck et al., 2018). Kappa agonistic drugs are the current recommendation for cockatiels based on studies in other psittacine species (Paul-Murphy et al., 1999, 2009a; Sladky et al., 2006); however, there was less *OPRK1* in the cerebrum compared to pigeons and less *OPRK1* compared to *OPRM1* in cockatiel tissues overall. This suggests that other pharmacokinetic or pharmacodynamic factors in addition to opioid receptor expression may be responsible for the varying analgesic response in avian species and should be investigated further.

Only two polymorphisms predicted to alter protein structure were identified. Both affected amino acid 221 in *OPRK1* in birds, corresponding to amino acid 131 or 220 in the *OPRK1* receptor in humans depending on the transcript. A missense variant at this position has been identified in humans (Ensembl rs1195432072), however no phenotype data was available and the effect is predicted to be benign by polyphen. This position is also poorly conserved across vertebrates (Supplementary Figure 2; Kent et al., 2002). Polymorphisms in opioid receptors could alter drug response (Kadiev et al., 2008). Although this variant is unlikely to influence gene expression, verifying its effect could be an avenue of future research.

Interestingly, there was decreased *OPRM1* expression in cockatiel footpad compared to pigeon footpad which supports a possible explanation for the lack of analgesic effect in cockatiels receiving mu-agonists during thermal foot withdrawal antinociceptive studies (Guzman et al., 2018; Houck et al., 2018). A large limitation of this study is a potential lack of power due to the small number of animals utilized. It is possible that some differences in opioid receptor distributions were not identified due to the limited sample size. Therefore, additional work with larger samples sizes is necessary to determine if the results found in this study are valid. If they are valid, future studies may aim to evaluate expression levels of *OPRM1* in other peripheral tissues used in antinociception studies.

Another possibility for observed differences in opioid response that was not addressed in this study was the presence of alternate transcripts of opioid receptors. A previous study in birds found that *OPRM1* had several different splicing transcripts that varied within and across species (Duhamelle et al., 2018). The reported splicing variants in avian species occurred at the 3′ end of the gene involving exons 3 and 4, but splicing variants at the 5′ end were not investigated (Duhamelle et al., 2018). In mice, rats, and humans there are splicing variants that lack exon 1 or exon 2 (Pasternak, 2014). This study used a single primer set contained within exon 2 to interrogate the expression of *OPRM1.* While this would allow the 3′ end splicing variants to be avoided, if splicing variants involving exon 2 are present in avian species, they would be missed by this study and likely lower the perceived expression levels of the receptor.

It is important to note that although opioid receptors are critical to the endogenous response to pain; they also participate in other normal physiological functions. However, the expression levels in those other tissues were not the focus of this study; therefore, the observed difference in level of expression for each receptor type does not distinguish its role in other diverse physiological functions.

It is possible that there are more detailed differences in opioid receptor gene expression that were not detected by this study. For example, in a separate study that used autoradiographic receptor binding techniques, there were differences in opioid distributions in specific regions of the pigeon brain (Reiner et al., 1989). Although efforts were made to take punch biopsies in repeatable, specific locations from each individual, gene expression differences may have been identified if finer dissections of the neurological opioid pathway were performed. For example, interrogating specific regions of the brain known to have a large role in analgesia such as the thalamus may have yielded species difference undetected by this study. Another limitation is that this study only evaluated two opioid receptors, *OPRM1* and *OPRK1*, and two avian species. It is conceivable that some of the variability in antinociceptive response may be due differential expression of other opioid receptors such as delta or differential expression in other species. An important limitation is that this study only looked at mRNA expression levels, not protein levels which could influence drug affinity for the receptors. More work is necessary to investigate the impact that the variation of gene expression has on opioid response.

## Conclusion

This preliminary study identified differential expression of mu and kappa opioid receptor genes within tissues of individual avian species and compared between two avian species. However, future research is necessary to determine if the expression differences between these two species occurs at a functional protein level, if these differences occur in other avian species, and if these differences influence analgesic responses.

## Data Availability Statement

All datasets presented in this study are included in the article/Supplementary Material.

## Ethics Statement

The animal study was reviewed and approved by the Institutional Animal Care and Use Committee at the University of California – Davis (IACUC #20331).

## Author Contributions

JS, JP-M, and DS-MG conceptualized and designed the study. SF, JP-M, and DS-MG performed the sample collection. SF and BG performed RNA extraction and qPCR. SF, BG, and JS performed statistical analysis. All authors contributed to writing, editing, and approval of submitted manuscript.

## Conflict of Interest

The authors declare that the research was conducted in the absence of any commercial or financial relationships that could be construed as a potential conflict of interest.

## Footnotes

1 Stuart’s Farm Fresh

## Abbreviations

ACTB: actin beta
Ct: cycle threshold
FDR: false discovery rate
GAPDH: glyceraldehyde-3-phosphate dehydrogenase
OPRM1: opioid receptor mu 1
OPRK1: opioid receptor kappa 1
PGK1: phosphoglycerate kinase 1
qPCR: quantitative real-time PCR.
