# Supplementary material for: Varying Expression of Mu and Kappa Opioid Receptors in Cockatiels (Nymphicus hollandicus) and Domestic Pigeons (Columba livia domestica)
Source: Front Genet. 2020 Oct 15;11:549558. doi: 10.3389/fgene.2020.549558 (PMC7593685; doi:10.3389/fgene.2020.549558)
Supplement: Supplementary file 2 [file Data_Sheet_2.docx]

**Supplemental Figure 1**. Specificity of primers in pigeons and cockatiels. Melting curves of five dilutions of pooled tissue samples from all individuals for each species run in triplicate. Gene names are in the panel.

**Supplemental Figure 2**. Basewise conservation of *OPRK1* missense variant illustrating poor conservation of the amino acid across vertebrates. Image obtained from <http://genome.ucsc.edu> using the Human Dec 2013 (GRCh38/hg38) assembly.

**Supplemental Table 1**. Opioid receptor polymorphisms with respect to the predicted *Columba livia* mRNA sequences

| Accession Number | Polymorphism | Individual | Predicted effect |
| --- | --- | --- | --- |
| XM_005507657.2 Predicted *Columba livia OPRM1* mRNA | c.154 T>C | All cockatiels | Synonymous |
| XM_005507657.2 Predicted *Columba livia OPRM1* mRNA | c. 169 C>T | All cockatiels | Synonymous |
| XM_005507657.2 Predicted *Columba livia OPRM1* mRNA | c. 181 G>A | All cockatiels | Synonymous |
| XM_005507657.2 Predicted *Columba livia OPRM1* mRNA | c.205 A>C | All cockatiels | Synonymous |
| XM_005507657.2 Predicted *Columba livia OPRM1* mRNA | c. 208 C>C/T | Cockatiels 3, 4, 7, and 8 | Synonymous |
| XM_005507657.2 Predicted *Columba livia OPRM1* mRNA | c. 220 C>T | All cockatiels | Synonymous |
| XM_005507657.2 Predicted *Columba livia OPRM1* mRNA | c. 238 T>C | All cockatiels | Synonymous |
| XM_005507657.2 Predicted *Columba livia OPRM1* mRNA | c. 265 A>G | All cockatiels | Synonymous |
| XM_005505451.3 Predicted *Columba livia OPRK1* mRNA | c. 1018 T>C | All cockatiels | Synonymous |
| XM_005505451.3 Predicted *Columba livia OPRK1* mRNA | c. 1105 C>T | All cockatiels | Synonymous |
| XM_005505451.3 Predicted *Columba livia OPRK1* mRNA | c. 1133 G>A | All cockatiels | Missense  p.Val221Met |
| XM_005505451.3 Predicted *Columba livia OPRK1* mRNA | c. 1135 G>G/T | Cockatiels 3, 4, and 6 | Missense  p.Val221Ile |

**Supplemental Table 2**. Significance of sex effect on gene expression for each tissue type and opioid receptor

|  | *OPRK1* p-values | *OPRM1* p-values |
| --- | --- | --- |
| Cockatiel Cerebrum | 0.66# | 0.60 |
| Pigeon Cerebrum | >0.99# | 0.91 |
| Cockatiel Brainstem | 0.11 | 0.62 |
| Pigeon Brainstem | 0.62 | 0.59 |
| Cockatiel Spinal Cord | 0.24 | 0.61 |
| Pigeon Spinal Cord | 0.78 | 0.77 |
| Cockatiel Footpad | 0.83 | 0.33 |
| Pigeon Footpad | 0.88 | 0.59 |

# Samples did not pass normality and a non-parametric Mann-Whitney test was used to determine significance

**Supplemental Table 3.** Delta Ct values for each tissue type, receptor, species, and sex

|  | Ct Value | Species  (Cockatiel=1,  Pigeon = 0) | Sex  (M=1, F=0) | Receptor (0=OPRK1, 1=OPRM1) | Tissue (0=cerebrum, 1=brainstem, 2=spinal cord, 3=footpad) |
| --- | --- | --- | --- | --- | --- |
| Pt 1 | -5.07 | 1 | 1 | 0 | 0 |
| Pt 2 | -2.18 | 1 | 1 | 0 | 0 |
| Pt 3 | -2.34 | 1 | 1 | 0 | 0 |
| Pt 4 | -1.34 | 1 | 1 | 0 | 0 |
| Pt 5 | -1.92 | 1 | 0 | 0 | 0 |
| Pt 6 | -2.30 | 1 | 0 | 0 | 0 |
| Pt 7 | -2.91 | 1 | 0 | 0 | 0 |
| Pt 8 | -0.37 | 1 | 1 | 0 | 0 |
| Pt 9 | -2.88 | 1 | 1 | 0 | 0 |
| Pt 10 | -5.67 | 1 | 0 | 0 | 0 |
| Pt 11 | -1.84 | 1 | 0 | 0 | 0 |
| Pg 1 | -1.94 | 0 | 0 | 0 | 0 |
| Pg 2 | -0.99 | 0 | 1 | 0 | 0 |
| Pg 3 | -0.84 | 0 | 0 | 0 | 0 |
| Pg 4 | -1.42 | 0 | 1 | 0 | 0 |
| Pg 5 | -0.85 | 0 | 1 | 0 | 0 |
| Pg 6 | -0.40 | 0 | 0 | 0 | 0 |
| Pg 7 | -1.25 | 0 | 1 | 0 | 0 |
| Pg 8 | 0.81 | 0 | 1 | 0 | 0 |
| Pg 9 | -0.54 | 0 | 0 | 0 | 0 |
| Pg 10 | -1.41 | 0 | 1 | 0 | 0 |
| Pg 11 | -1.76 | 0 | 0 | 0 | 0 |
| Pt 1 | 0.89 | 1 | 1 | 1 | 0 |
| Pt 2 | 1.06 | 1 | 1 | 1 | 0 |
| Pt 3 | 0.03 | 1 | 1 | 1 | 0 |
| Pt 4 | 1.25 | 1 | 1 | 1 | 0 |
| Pt 5 | -0.23 | 1 | 0 | 1 | 0 |
| Pt 6 | 1.23 | 1 | 0 | 1 | 0 |
| Pt 7 | 1.36 | 1 | 0 | 1 | 0 |
| Pt 8 | 1.43 | 1 | 1 | 1 | 0 |
| Pt 9 | 0.44 | 1 | 1 | 1 | 0 |
| Pt 10 | 1.25 | 1 | 0 | 1 | 0 |
| Pt 11 | -0.57 | 1 | 0 | 1 | 0 |
| Pg 1 | -1.19 | 0 | 0 | 1 | 0 |
| Pg 2 | 1.97 | 0 | 1 | 1 | 0 |
| Pg 3 | 2.61 | 0 | 0 | 1 | 0 |
| Pg 4 | 1.23 | 0 | 1 | 1 | 0 |
| Pg 5 | 1.28 | 0 | 1 | 1 | 0 |
| Pg 6 | 1.17 | 0 | 0 | 1 | 0 |
| Pg 7 | -0.35 | 0 | 1 | 1 | 0 |
| Pg 8 | 0.64 | 0 | 1 | 1 | 0 |
| Pg 9 | 1.81 | 0 | 0 | 1 | 0 |
| Pg 10 | 0.50 | 0 | 1 | 1 | 0 |
| Pg 11 | -0.42 | 0 | 0 | 1 | 0 |
| Pt 1 | -5.26 | 1 | 1 | 0 | 1 |
| Pt 2 | -7.56 | 1 | 1 | 0 | 1 |
| Pt 3 | -6.92 | 1 | 1 | 0 | 1 |
| Pt 4 | -5.29 | 1 | 1 | 0 | 1 |
| Pt 5 | -4.78 | 1 | 0 | 0 | 1 |
| Pt 6 | -3.14 | 1 | 0 | 0 | 1 |
| Pt 7 | -5.11 | 1 | 0 | 0 | 1 |
| Pt 8 | -5.06 | 1 | 1 | 0 | 1 |
| Pt 9 | -3.49 | 1 | 1 | 0 | 1 |
| Pt 10 | -4.42 | 1 | 0 | 0 | 1 |
| Pt 11 | -4.17 | 1 | 0 | 0 | 1 |
| Pg 1 | -7.40 | 0 | 0 | 0 | 1 |
| Pg 2 | -9.31 | 0 | 1 | 0 | 1 |
| Pg 3 | -11.01 | 0 | 0 | 0 | 1 |
| Pg 4 | -6.96 | 0 | 1 | 0 | 1 |
| Pg 5 | -5.40 | 0 | 1 | 0 | 1 |
| Pg 6 | -4.30 | 0 | 0 | 0 | 1 |
| Pg 7 | -5.20 | 0 | 1 | 0 | 1 |
| Pg 8 | -3.76 | 0 | 1 | 0 | 1 |
| Pg 9 | -3.52 | 0 | 0 | 0 | 1 |
| Pg 10 | -2.24 | 0 | 1 | 0 | 1 |
| Pg 11 | -5.36 | 0 | 0 | 0 | 1 |
| Pt 1 | 0.19 | 1 | 1 | 1 | 1 |
| Pt 2 | 0.32 | 1 | 1 | 1 | 1 |
| Pt 3 | 0.46 | 1 | 1 | 1 | 1 |
| Pt 4 | 2.70 | 1 | 1 | 1 | 1 |
| Pt 5 | 0.71 | 1 | 0 | 1 | 1 |
| Pt 6 | 3.35 | 1 | 0 | 1 | 1 |
| Pt 7 | 1.55 | 1 | 0 | 1 | 1 |
| Pt 8 | 0.99 | 1 | 1 | 1 | 1 |
| Pt 9 | 3.01 | 1 | 1 | 1 | 1 |
| Pt 10 | 0.67 | 1 | 0 | 1 | 1 |
| Pt 11 | 2.00 | 1 | 0 | 1 | 1 |
| Pg 1 | -0.40 | 0 | 0 | 1 | 1 |
| Pg 2 | 0.48 | 0 | 1 | 1 | 1 |
| Pg 3 | 1.33 | 0 | 0 | 1 | 1 |
| Pg 4 | -0.70 | 0 | 1 | 1 | 1 |
| Pg 5 | 1.12 | 0 | 1 | 1 | 1 |
| Pg 6 | 1.04 | 0 | 0 | 1 | 1 |
| Pg 7 | -0.52 | 0 | 1 | 1 | 1 |
| Pg 8 | -0.21 | 0 | 1 | 1 | 1 |
| Pg 9 | 1.06 | 0 | 0 | 1 | 1 |
| Pg 10 | 2.05 | 0 | 1 | 1 | 1 |
| Pg 11 | 0.39 | 0 | 0 | 1 | 1 |
| Pt 1 | -1.81 | 1 | 1 | 0 | 2 |
| Pt 2 | -3.48 | 1 | 1 | 0 | 2 |
| Pt 3 | -4.14 | 1 | 1 | 0 | 2 |
| Pt 4 | -3.71 | 1 | 1 | 0 | 2 |
| Pt 5 | -5.64 | 1 | 0 | 0 | 2 |
| Pt 6 | -4.18 | 1 | 0 | 0 | 2 |
| Pt 7 | -2.32 | 1 | 0 | 0 | 2 |
| Pt 8 | -3.23 | 1 | 1 | 0 | 2 |
| Pt 9 | -3.58 | 1 | 1 | 0 | 2 |
| Pt 10 | -3.69 | 1 | 0 | 0 | 2 |
| Pt 11 | -4.68 | 1 | 0 | 0 | 2 |
| Pg 1 | -2.83 | 0 | 0 | 0 | 2 |
| Pg 2 | -1.53 | 0 | 1 | 0 | 2 |
| Pg 3 | -2.67 | 0 | 0 | 0 | 2 |
| Pg 4 | -3.77 | 0 | 1 | 0 | 2 |
| Pg 5 | -2.74 | 0 | 1 | 0 | 2 |
| Pg 6 | -2.17 | 0 | 0 | 0 | 2 |
| Pg 7 | -3.67 | 0 | 1 | 0 | 2 |
| Pg 8 | -3.62 | 0 | 1 | 0 | 2 |
| Pg 9 | -4.55 | 0 | 0 | 0 | 2 |
| Pg 10 | -3.00 | 0 | 1 | 0 | 2 |
| Pg 11 | -3.86 | 0 | 0 | 0 | 2 |
| Pt 1 | 2.32 | 1 | 1 | 1 | 2 |
| Pt 2 | 1.52 | 1 | 1 | 1 | 2 |
| Pt 3 | 1.23 | 1 | 1 | 1 | 2 |
| Pt 4 | 0.99 | 1 | 1 | 1 | 2 |
| Pt 5 | 0.82 | 1 | 0 | 1 | 2 |
| Pt 6 | 0.75 | 1 | 0 | 1 | 2 |
| Pt 7 | 0.00 | 1 | 0 | 1 | 2 |
| Pt 8 | 2.00 | 1 | 1 | 1 | 2 |
| Pt 9 | 2.44 | 1 | 1 | 1 | 2 |
| Pt 10 | 2.65 | 1 | 0 | 1 | 2 |
| Pt 11 | 2.97 | 1 | 0 | 1 | 2 |
| Pg 1 | 0.80 | 0 | 0 | 1 | 2 |
| Pg 2 | 2.64 | 0 | 1 | 1 | 2 |
| Pg 3 | 1.27 | 0 | 0 | 1 | 2 |
| Pg 4 | 1.53 | 0 | 1 | 1 | 2 |
| Pg 5 | 3.23 | 0 | 1 | 1 | 2 |
| Pg 6 | 2.38 | 0 | 0 | 1 | 2 |
| Pg 7 | 2.35 | 0 | 1 | 1 | 2 |
| Pg 8 | 1.76 | 0 | 1 | 1 | 2 |
| Pg 9 | 2.66 | 0 | 0 | 1 | 2 |
| Pg 10 | 2.76 | 0 | 1 | 1 | 2 |
| Pg 11 | 3.91 | 0 | 0 | 1 | 2 |
| Pt 1 | -8.86 | 1 | 1 | 0 | 3 |
| Pt 2 | -11.31 | 1 | 1 | 0 | 3 |
| Pt 3 | -10.65 | 1 | 1 | 0 | 3 |
| Pt 4 | -10.91 | 1 | 1 | 0 | 3 |
| Pt 5 | -9.80 | 1 | 0 | 0 | 3 |
| Pt 6 |  | 1 | 0 | 0 | 3 |
| Pt 7 | -10.02 | 1 | 0 | 0 | 3 |
| Pt 8 | -11.51 | 1 | 1 | 0 | 3 |
| Pt 9 | -10.99 | 1 | 1 | 0 | 3 |
| Pt 10 | -11.52 | 1 | 0 | 0 | 3 |
| Pt 11 | -10.96 | 1 | 0 | 0 | 3 |
| Pg 1 |  | 0 | 0 | 0 | 3 |
| Pg 2 | -10.83 | 0 | 1 | 0 | 3 |
| Pg 3 | -11.36 | 0 | 0 | 0 | 3 |
| Pg 4 | -11.44 | 0 | 1 | 0 | 3 |
| Pg 5 | -11.21 | 0 | 1 | 0 | 3 |
| Pg 6 | -9.88 | 0 | 0 | 0 | 3 |
| Pg 7 | -10.90 | 0 | 1 | 0 | 3 |
| Pg 8 | -10.87 | 0 | 1 | 0 | 3 |
| Pg 9 | -9.95 | 0 | 0 | 0 | 3 |
| Pg 10 | -10.60 | 0 | 1 | 0 | 3 |
| Pg 11 | -12.42 | 0 | 0 | 0 | 3 |
| Pt 1 | -4.42 | 1 | 1 | 1 | 3 |
| Pt 2 | -6.98 | 1 | 1 | 1 | 3 |
| Pt 3 | -8.48 | 1 | 1 | 1 | 3 |
| Pt 4 | -7.91 | 1 | 1 | 1 | 3 |
| Pt 5 | -5.17 | 1 | 0 | 1 | 3 |
| Pt 6 | -5.18 | 1 | 0 | 1 | 3 |
| Pt 7 | -6.79 | 1 | 0 | 1 | 3 |
| Pt 8 | -5.29 | 1 | 1 | 1 | 3 |
