# Supplementary figures and images for: Varying Expression of Mu and Kappa Opioid Receptors in Cockatiels (Nymphicus hollandicus) and Domestic Pigeons (Columba livia domestica)
Source: Front Genet. 2020 Oct 15;11:549558. doi: 10.3389/fgene.2020.549558 (PMC7593685; doi:10.3389/fgene.2020.549558)

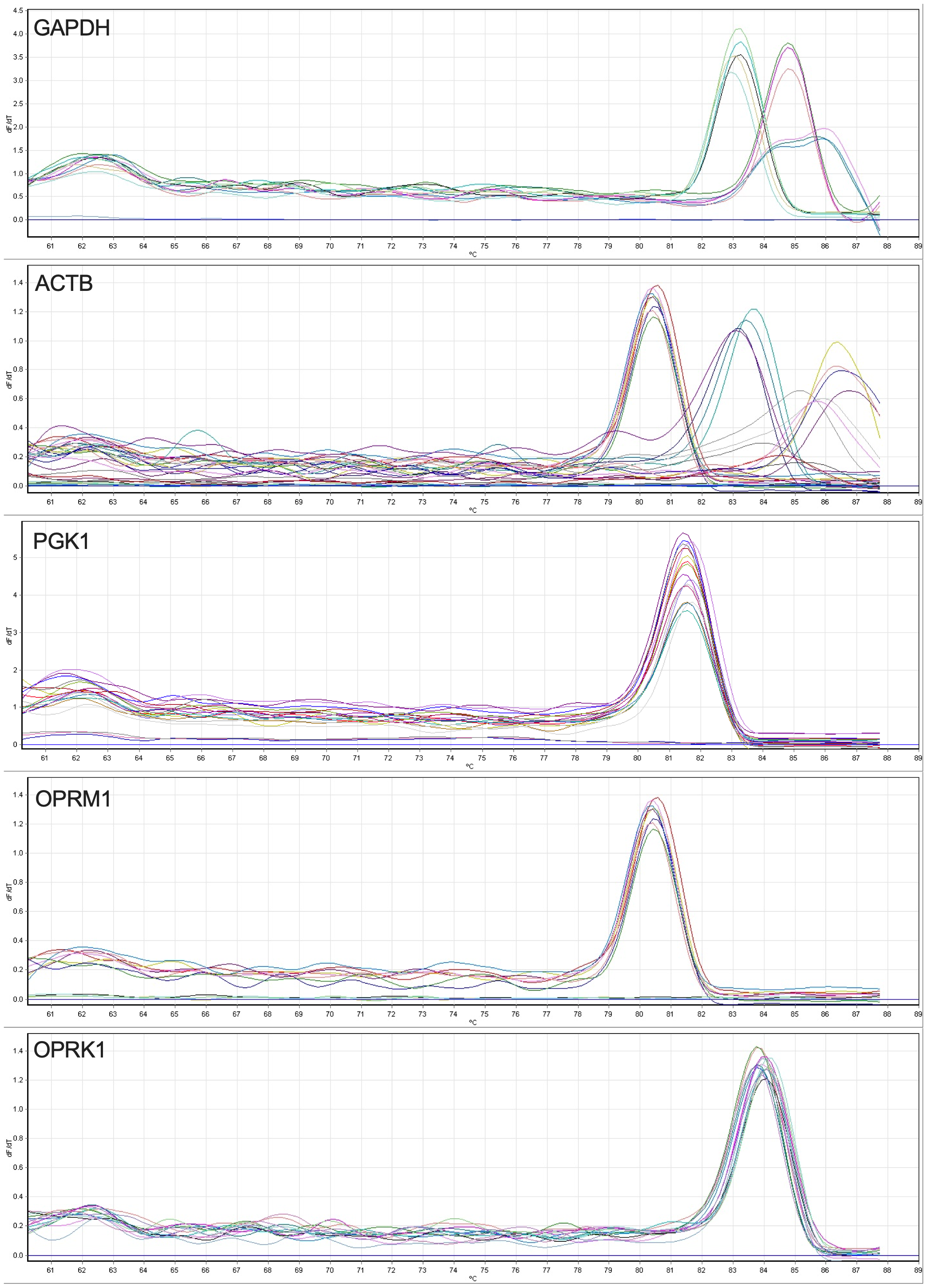

Supplement: Supplementary file 3 [file Image_1.TIFF]

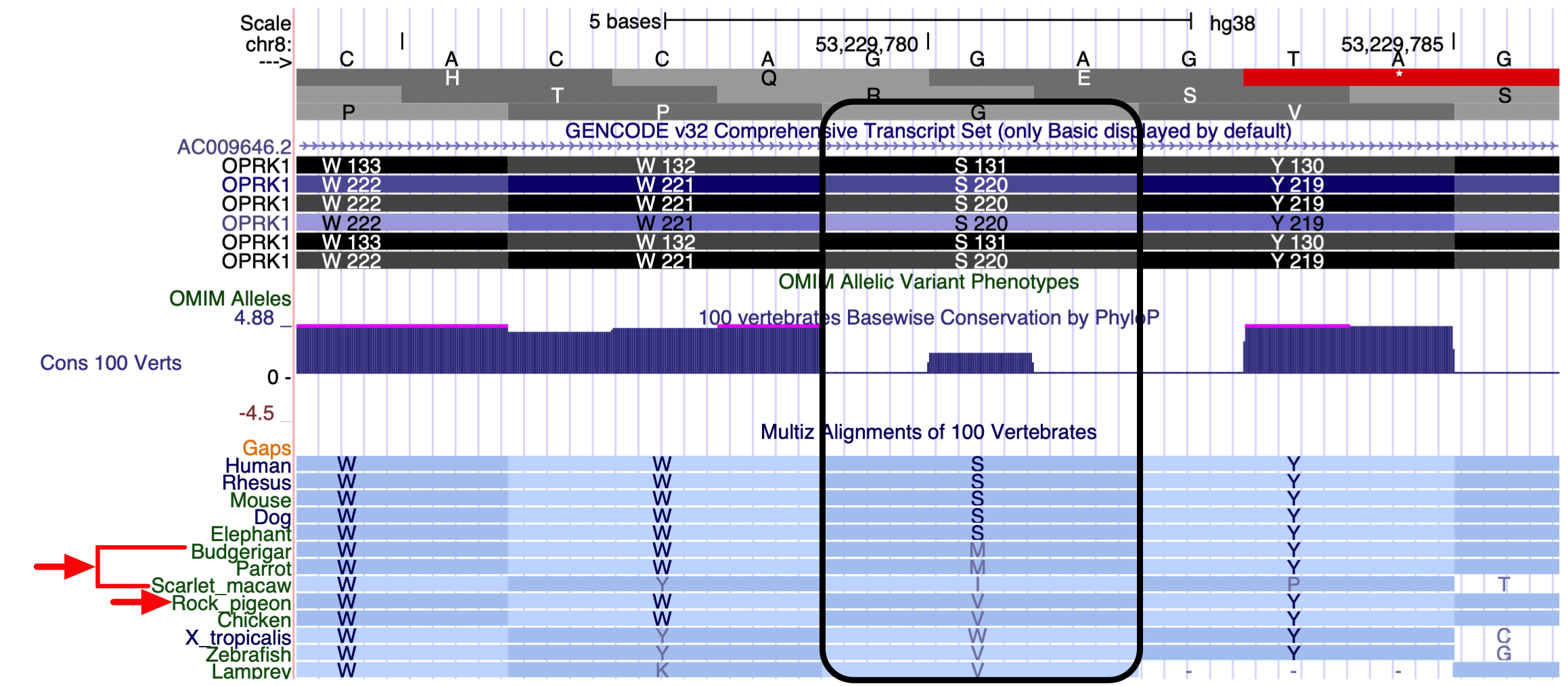

Supplement: Supplementary file 4 [file Image_2.TIFF]
